# Ectomycorrhizal fungi and the nitrogen economy of *Nothofagus* in southern Patagonia

**DOI:** 10.1002/ece3.70299

**Published:** 2024-09-29

**Authors:** Camille Truong, Luciano A. Gabbarini, Alicia Moretto, Julio M. Escobar, Matthew E. Smith

**Affiliations:** ^1^ Royal Botanic Gardens Victoria Melbourne Victoria Australia; ^2^ Departamento de Ciencia y Tecnología, Centro de Bioquímica y Microbiología de Suelos Universidad Nacional de Quilmes Bernal Argentina; ^3^ Universidad Nacional de Tierra del Fuego, Instituto de Ciencias Polares, Recursos Naturales y Ambiente Ushuaia Argentina; ^4^ Centro Austral de Investigaciones Científicas (CONICET) Ushuaia Argentina; ^5^ Department of Plant Pathology University of Florida Gainesville Florida USA

**Keywords:** leaf phenology, mycorrhizal associations, Nothofagaceae, nutrient cycling, soil fertility, southern hemisphere, subantarctic forests

## Abstract

Subantarctic *Nothofagus* forests are the southernmost forests in the world, with negligible atmospheric nitrogen (N) deposition. Most paradigms about the role of ectomycorrhizal (ECM) fungi in N cycling and plant N uptake at high latitudes have been tested in boreal coniferous forests, while in the southern hemisphere, ECM hosts are primarily angiosperms. Using ITS1 meta‐barcoding, we characterized ECM and saprotrophic fungal communities in evergreen and deciduous *Nothofagus* forests forming monodominant and mixed stands in the archipelago of Tierra del Fuego (Chile and Argentina). We assessed the N economy of *Nothofagus* by correlating host species with fungal relative abundances, edaphic variables, net N mineralization, microbial biomass N and the activity of eight extracellular soil enzymes activities. The N economy of deciduous *N. pumilio* forests was strikingly similar to boreal coniferous forests, with the lowest inorganic N availability and net N mineralization, in correlation to higher relative abundances of ECM fungi with enzymatic capacity for organic N mobilization (genus *Cortinarius*). In contrast, the N economy of evergreen *N. betuloides* forests was predominantly inorganic and correlated with ECM lineages from the family Clavulinaceae, in acidic soils with poor drainage. Grassy understory vegetation in deciduous *N. antarctica* forests likely promoted saprotrophic fungi (i.e., genus *Mortierella*) in correlation with higher activities of carbon‐degrading enzymes. Differences between *Nothofagus* hosts did not persist in mixed forests, illustrating the range of soil fertility of these ECM angiosperms and the underlying effects of soil and climate on *Nothofagus* distribution and N cycling in southern Patagonia.

## Graphical abstract

Ectomycorrhizal fungi play essential roles in nitrogen cycling and plant nutrient uptake at high latitudes. However, they have disproportionately been studied in boreal forests dominated by evergreen conifers. We characterized ectomycorrhizal associations and the nitrogen economy of co‐occurring deciduous and evergreen angiosperms from the genus *Nothofagus* across fertility gradients in Patagonia. Our findings illustrate the diversity of nitrogen acquisition strategies of ECM fungi in these forests that do not always reflect northern hemisphere paradigms.

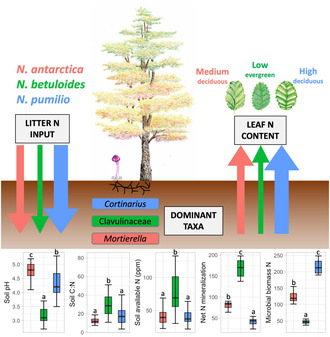

## Resumen

Los bosques subantárticos de *Nothofagus* son los más australes del mundo, en donde la deposición atmosférica de nitrógeno (N) es casi nula. La mayoría de los estudios sobre el papel de hongos ectomicorrícicos (ECM) está basado en bosques de coníferas perennes, en el hemisferio norte; mientras que en el hemisferio sur los hospederos ECM son principalmente angiospermas. Caracterizamos las comunidades de hongos ECM y saprobios del suelo por secuenciación de ADN ambiental de la región ITS1 de 150 muestras recolectadas en bosques perennes, caducifolios y mixtos dominados por *Nothofagus* en el archipiélago de Tierra del Fuego (Argentina y Chile). Estudiamos la economía del N en diferentes bosques de *Nothofagus*, evaluando las correlaciones entre las comunidades de hongos ECM y saprobios con sus hospederos, variables edáficas, mineralización neta del N, biomasa microbiana del N y la actividad de ocho enzimas extracelulares. Encontramos que la economía del N de los bosques caducifolios de *N. pumilio* es sorprendentemente similar a la de los bosques de coníferas boreales, en los que encontramos los valores más bajos de N orgánico disponible y de mineralización neta del N, en correlación con mayor abundancia relativa de hongos ECM con capacidad enzimáticas de descomposición (género *Cortinarius*). En contraste, la economía del N de los bosques perennes de *N. betuloides* es predominantemente inorgánica y se correlaciona con abundancia alta de hongos ECM de la familia Clavulinaceae, preferentemente en suelos ácidos y anegadizos. La vegetación herbácea del sotobosque en los bosques caducifolios de *N. antarctica* posiblemente ha aumentado la abundancia de hongos saprobios (p. ej., *Mortierella*) que se correlaciona con una mayor actividad de enzimas de degradación del carbono. Las diferencias encontradas entre bosques perennes y caducifolios no persistieron en los bosques mixtos, lo que ilustra el gradiente de fertilidad del suelo de estas angiospermas formadoras de ectomicorrizas, y los efectos subyacentes del suelo y el clima en la distribución de *Nothofagus* y el ciclo del N en la región.

## 1 INTRODUCTION

Nitrogen (N) is an essential nutrient for tree growth and forest productivity (Etzold et al., 2020; LeBauer & Treseder, 2008). The N economy of forest trees is determined by a variety of interacting factors, including the physiological N uptake capacities of the plant, the amount and nature of soil organic/inorganic N, and site‐adapted communities of mycorrhizal symbionts (Kranabetter, 2014). Leaf phenology can affect the N economy of trees, with consequences for plant–soil feedbacks and N cycling in forest soils: Compared to deciduous trees, evergreen species tend to exhibit a higher leaf lifespan, lower N and P contents, and lower litter decomposition rates, owing to greater litter recalcitrance (Reich & Oleksyn, 2004). Subsequently, soils under evergreen trees typically have a lower pH and slower N cycling rates than those under deciduous trees (Mueller et al., 2012; Ordoñez et al., 2009).

Ectomycorrhizal (ECM) fungi are major components of forest soils at high latitudes (Steidinger et al., 2019). They provide their host with essential nutrients, particularly N, in exchange for a carbon (C) source (Read & Perez‐Moreno, 2003). N bound in soil organic matter (SOM) is by far the largest N pool in soils, but was historically considered inaccessible to plants that mostly absorb inorganic N available via microbial mineralization (Schimel & Bennett, 2004). Recent studies demonstrated that some ECM fungal groups, such as *Cortinarius*, possess enzymatic pathways to mine N from SOM, and directly compete with free‐living saprotrophs (SAP) for access to organic substrates (Lindahl & Tunlid, 2015; Sterkenburg et al., 2018). These mechanisms allow their host plants to “short‐circuit” inorganic N cycling, therefore, favoring ECM fungal groups with organic N mining capabilities, as N availability declines (Corrales et al., 2017; Pellitier & Zak, 2021). N‐limited conditions are particularly ubiquitous in coniferous boreal forests, where ECM fungi account for one‐third of the total microbial biomass in soils (Högberg & Högberg, 2002). These forests are particularly sensitive to anthropogenic N deposition that can potentially induce shifts toward tree species forming other types of mycorrhizal associations (Etzold et al., 2020; Jo et al., 2019; Mao et al., 2019). However, the impact of ECM fungi on plant nutrition and C/N pools is context‐dependent and ECM fungal communities have been documented across a wide range of soil fertility, for example, in temperate rainforests of North America (McPolin et al., 2024; Pellitier & Zak, 2021).

Since ECM forests of the northern hemisphere are dominated by conifers (Brundrett & Tedersoo, 2020), most studies on plant–soil feedback involving leaf phenology have compared evergreen conifers with deciduous angiosperms (Midgley & Sims, 2020). In contrast, ECM forests of the southern hemisphere are dominated by angiosperms, but ECM associations in these ecosystems remain heavily understudied (Nouhra et al., 2019). Patagonia is one of the most unpolluted regions of the world, with negligible atmospheric N deposition (Perakis & Hedin, 2001). The region is extensively covered by Andean‐Patagonian forests on both sides of the Andes (Chile and Argentina) from latitudes 35° to 55°, which makes them the southernmost forests in the world (Buma et al., 2021). They are geographically isolated from other forests in South America since the Oligocene (23–33 MYA), resulting in high levels of endemism despite their low plant species richness (Marchelli et al., 2020). Andean‐Patagonian forests are dominated by ECM angiosperms in the family Nothofagaceae that provide a wide range of ecological, economic and social benefits (Mattera et al., 2020). Nothofagaceae have been the only native ECM hosts in Patagonia for at least 50 MY (Gandolfo et al., 2011) and many of their ECM fungal symbionts are endemic to the southern hemisphere (Tedersoo et al., 2010; Truong et al., 2017).

Andean‐Patagonian forests are traditionally divided into temperate rainforests and subantarctic forests (South of latitude 47°), the latter being more species‐poor (Moreira‐Muñoz, 2011). Soils in subantarctic forests of Tierra del Fuego are of glacial origin, while further north in temperate rainforests, they originate from volcanic ashes (Godoy et al., 2013). Subantarctic forests are composed of monodominant stands of deciduous and evergreen *Nothofagus* species distributed along soil fertility gradients (Diehl et al., 2008; Romanyà et al., 2005). Contrary to Northern hemisphere forests, forests dominated by arbuscular mycorrhizal (AM) associations are absent from the region. Thébault et al. (2014) suggested that low nutrient availability limited *Nothofagus* growth at the treeline, because of competition for N between trees and soil microbes. ECM fungal diversity also correlated negatively with N availability in *N. pumilio* forests, as host trees tend to reduce C allocation to their root symbionts when there is an excess of readily available N (Truong et al., 2019). *Cortinarius* species are hyper‐diverse and abundant in Patagonian forests (Truong et al., 2017) and likely contribute to plant access to organic N sources. Other actors, such as ericoid mycorrhizal fungi associated with understory vegetation (Ward et al., 2022) likely play a role, but the functions of soil fungal communities for C and N cycling remain largely unknown in South America (Nouhra et al., 2019).

Here, we used ITS1 metabarcoding to characterize soil fungal communities in monodominant and mixed *Nothofagus* forests in the archipelago of Tierra del Fuego in southern Patagonia (Argentina and Chile). We tested the correlations between ECM or SAP fungal communities and *Nothofagus* host species with different leaf phenology (evergreen *N. betuloides* and deciduous *N. antarctica* and *N. pumilio*), as well as edaphic variables, net N mineralization, microbial biomass N, and the activity of eight extracellular soil enzymes. We hypothesized that (i) N mineralization and N availability will be lower in evergreen *N. betuloides* forest than in deciduous *N. antarctica* and *N. pumilio* forests; (ii) ECM fungi with enzymatic capacity for organic N mobilization will negatively correlate with soil N availability and N mineralization, similarly to patterns observed in coniferous boreal forests; and (iii) mixed forests will show intermediate edaphic conditions, with fewer differences in ECM fungal community composition and enzyme activities between *Nothofagus* hosts.

## 2 MATERIALS AND METHODS

### 2.1 Focal species

**FIGURE 1:**
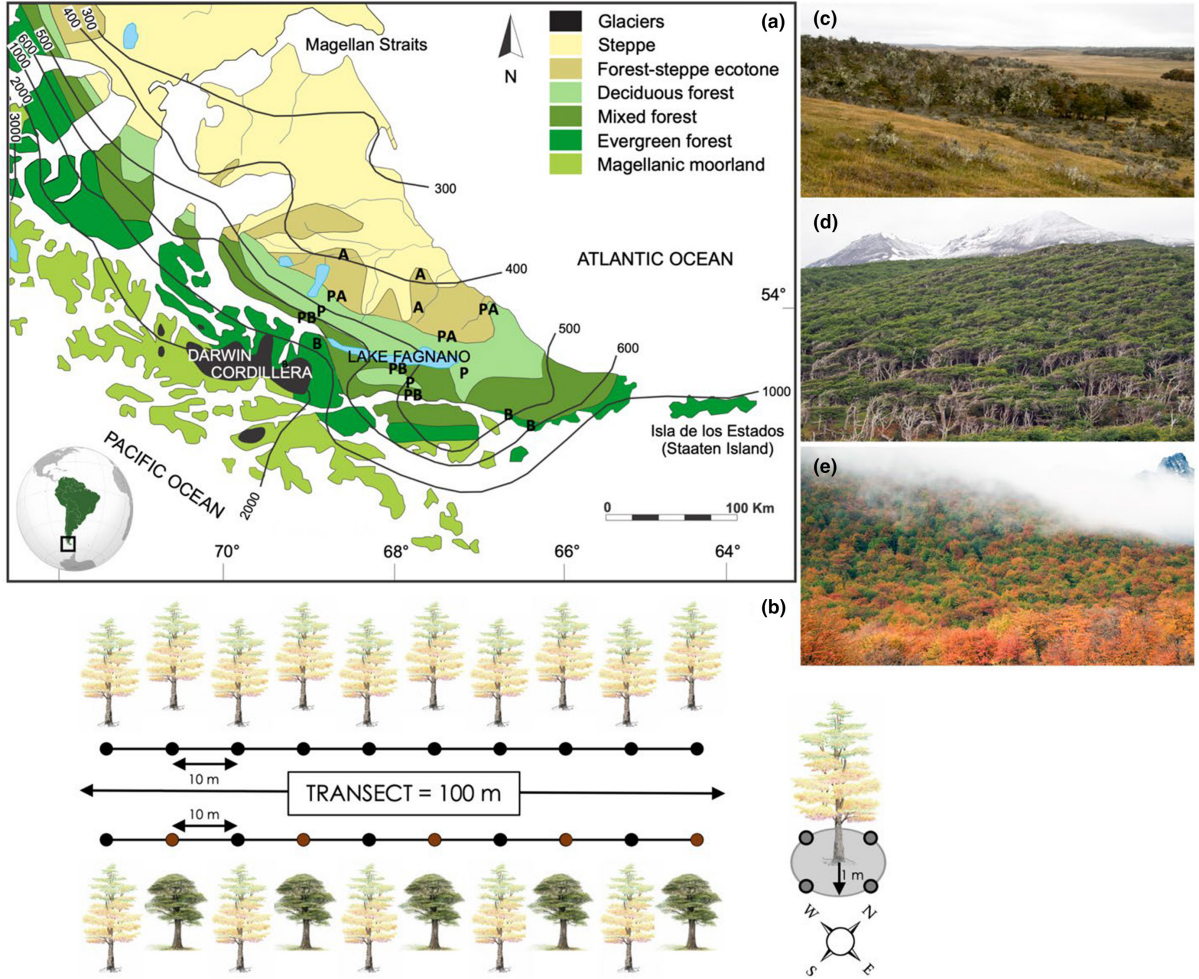
(a) Vegetation map of Tierra del Fuego (modified from Musotto et al., 2017, used with permission) with mean annual precipitation isohyets and sampling plots indicated with letters: A = *N. antarctica*, B = *N. betuloides*, P = *N. pumilio*, PA = mixed *pumilio‐antarctica*, PB = *mixed pumilio‐betuloides*. (b) Sampling scheme in monodominant and mixed forests along a 100 m transect; for each sample, four soil cores were collected beneath each focal tree in cardinal directions. (c) Deciduous *N. antarctica* forest in the ecotone with the Patagonian steppe in the northern part of Tierra del Fuego. (d) Evergreen *N. betuloides* forest along the coast of the Beagle channel. (e) Deciduous *N. pumilio* forest along mountain slopes of the Andes cordillera in falls.

The archipelago of Tierra del Fuego lies at the southern tip of South America at latitudes 52.5–56° S. Mean annual temperatures at sea level range between 5 and 8°C, while mean annual precipitation varies from 300 mm in the north to >4000 mm in south‐western islands (Frangi et al., 2005). Old growth *Nothofagus* forests represent >45% of the tree cover in the archipelago and have experienced minimal anthropogenic perturbations (Global Forest Watch, 2014). *Nothofagus* is the only ECM host in Tierra del Fuego and no AM‐dominant forest occur in the region. Three *Nothofagus* species occur in Tierra del Fuego and form monodominant forests that are well separated across edaphic and climatic gradients created by the proximity of the Andes to the Atlantic and Pacific oceans (Figure 1, Musotto et al., 2017).

The deciduous *N. antarctica* is a stress‐tolerant species distributed throughout Patagonia in sites with an array of limiting factors for plant growth, such as drought, poor drainage, or freezing temperatures (Dettmann et al., 2013; Peri et al., 2009). In Tierra del Fuego it is most abundant along an ecotone with lowland Patagonian steppe in the northern continental regions that receive less precipitation (<450 mm/year); soils are mollisols‐haploxerolls with enhanced SOM decomposition (Bahamonde et al., 2012). Grasslands that spontaneously grow under the open *N. antarctica* canopy are naturally grazed by native guanacos and are frequently used for silvopastoral agriculture (Peri et al., 2016).

The evergreen *N. betuloides* is the southernmost tree in the world, growing as far as latitude 56° (Buma et al., 2021). In Tierra del Fuego, it is distributed along the coast and major lakes from sea level to ca. 350 m a.s.l., in oceanic sites that receive high rainfall (>600 mm/year); soils are shallow, periodically waterlogged, and highly acidic from the accumulation of recalcitrant SOM (Romanyà et al., 2005). *N. betuloides* can form dense monodominant forests (>80% basal area), with few other sparsely distributed trees or shrubs, such as *Drimys winteri* and *Maytenus magellanica*; the understory is sparse (ca. 30% bare soil cover) and dominated by mosses, some ferns and vascular plants (Mestre et al., 2017; Promis et al., 2008).

The deciduous *N. pumilio* is widely distributed in Patagonia (Marchelli et al., 2020). In Tierra del Fuego, it is distributed in the central mountain range (150–750 m a.s.l) where it forms monospecific forests (100% basal area), with a sparse understory (ca. 15% bare soil cover) dominated by herbaceous species, such as *Dysopsis glechomoides*, *Gavilea lutea* and *Viola magellanica* (Mestre et al., 2017; Rosas et al., 2019). Soils are well drained and characterized by podzolization (Romanyà et al., 2005), in sites with a wider temperature range and lower rainfall (<500 mm/year) than in *N. betuloides* forests. Based on its higher N and P leaf content, it was suggested that *N. pumilio* has higher N demands than *N. antarctica and N. betuloides* and therefore grows on more fertile soils (Diehl et al., 2008; Romanyà et al., 2005), but these assumptions have never been tested. *Nothofagus pumilio* co‐occur with *N. betuloides* in a narrow transition zone between coastal evergreen forests and deciduous forests in the interior, as well as with *N. antarctica* in a mosaic of grasslands and woodlands at the forest‐steppe ecotone (Frangi et al., 2005).

### 2.2 Rhizosphere soil sampling

Between February and March 2015, we established three plots in each of the following *Nothofagus* forests (Figure 1a): in monodominant stands of *N. antarctica* (50–150 m a.s.l.), *N. betuloides* (10–150 m a.s.l.), and *N. pumilio* (130–250 m a.s.l.), as well as in mixed *pumilio‐antarctica* (120–170 m a.s.l.), and *pumilio‐betuloides* forests (120–250 m a.s.l.), for a total of 15 plots. Mixed stands of *antarctica‐betuloides* do not exist because these two species do not overlap in their distributions. All plots were established in old‐growth forests unaffected by recent logging, fire, or silvopastoral farming. At each plot, we collected rhizosphere soil beneath one tree every 10 m along a 100 m transect, for a total of 10 individuals per plot (*n* = 150 samples, Figure 1b). In mixed forests, soil samples were collected beneath five trees of each host species at least 10 m apart. Each sample was composed of four soil cores (5 cm diam. × 10 cm deep, including upper mineral soil and organic horizons) after removing the litter, at the base of each individual tree in cardinal directions. Samples were maintained at <10°C and processed within 24 h.

### 2.3 Edaphic variables

We calculated percent soil moisture after drying 2.5–5 g of fresh sieved soil at 60°C for 48 h. Air dried soil was used to characterize: (i) pH in KCl 1 M (1:10); (ii) total C by dry combustion; (iii) total N by semi‐micro Kjeldahl (Bremner, 1996); (iv) concentration of NO_3_^−^ and NH_4_^+^ (Keeney & Nelson, 1982); and (v) available phosphorus (P) with the Bray & Kurtz 1 method (Kuo, 1996). NO_3_^−^ and NH_4_^+^ were summed into available N. Additionally, net N mineralization and microbial biomass N were measured in five samples randomly chosen from each monodominant forest plot, for a total of 45 samples. Net N mineralization was estimated as the difference in NH_4_–N + NO_3_–N after 28 days incubation in 250 mL plastic boxes at 25°C, in aerobic conditions and field water capacity, using a randomized design (Mazzarino et al., 1991). Field water capacity was controlled weekly by gravimetry, and the boxes were left exposed to air for 1 h. Microbial biomass N was determined in 50 g subsamples using a modification of the chloroform fumigation‐incubation technique (Mazzarino et al., 1991; Vitousek & Matson, 1985): Briefly, 1 mL of chloroform was added to each soil sample which was then incubated for 10 days at room temperature after chloroform had dissipated. N retained in microbial biomass was determined as the ammonium difference between day 0 and day 10. Fumigated and non‐fumigated samples were kept at field water capacity and the data were corrected for dry weight.

### 2.4 Extracellular soil enzyme activities

A total of 5–10 g of fresh sieved rhizosphere soil was maintained at 4°C for <1 week before measuring the activity of eight extracellular soil enzymes using fluorogenic substrates (Sigma‐Aldrich, St. Louis, MO, USA): Five C‐acquiring enzymes α‐glucosidase (AGLU), β‐glucosidase (BGLU), β‐glucuronidase (GLUCU), β‐xylosidase (XIL) and cellobiohydrolase (CEL), two N‐acquiring enzymes leucine aminopeptidase (LEU), and N‐acetyl‐glucosaminidase (NAG), and one P‐acquiring enzyme acid phosphatase (PHOS). Briefly, fluorescence intensity was read with an excitation of 355 nm and an emission of 460 nm on a POLARstar Omega computerized microplate fluorimeter (BMG LABTECH, Ortenberg, Germany) following the protocols detailed in Truong et al. (2019). Enzymatic activities were calculated based on three replicates per sample and expressed as nmol h^−1^ g^−1^.

### 2.5 ITS1 soil metabarcoding

Methods follow the protocols of Truong et al. (2019). Briefly, DNA was extracted from approx. 0.25 g of soil using the PowerSoil DNA Isolation kit and purified with the PowerClean Pro Clean‐Up kit (MO BIO, Carlsbad, CA, USA). The ITS1 rDNA region was amplified by PCR with primers ITS1f/ITS2, normalized at equimolar concentration with the SequalPrep Normalization Plate Kit (ThermoFisher Scientific, Waltham, MA, USA) and sequenced with a MiSeq 300 bp paired‐end protocol (Illumina, San Diego, CA, USA) at the Interdisciplinary Center for Biotechnology Research (ICBR) at the University of Florida. Raw sequence data are available at NCBI's Sequence Read Archive, Bioproject PRJNA476118. Quality filtering was performed in Trimmomatic (Bolger et al., 2014) and singleton sequences were filtered out. ITS1 fungal sequences were extracted with ITSx (Bengtsson‐Palme et al., 2013). Chimera filtering and clustering into operational taxonomic units (OTU) at 97% similarity was conducted with *usearch61* in QIIME 1.9.1 (Caporaso et al., 2010) by successively grouping (i) merged reads, (ii) unmerged forward reads because ITS1 was too long, (iii) unmerged forward reads for which the complementary sequence did not pass quality filtering, and (iv) unmerged reverse reads for which the complementary sequence did not pass quality filtering. This approach proved effective to retrieve fungal groups that may otherwise remain undetected due to the length of ITS1 or the low read quality in one direction (Truong et al., 2019). We used negative and positive controls, and OTU occurrences that accounted for <0.5% of the total read count per sample were removed to eliminate potential sequencing artifacts (Tedersoo et al., 2022). OTU taxonomy was assigned by performing BLASTn searches in QIIME and manually with MegaBLAST searches in PlutoF (Abarenkov et al., 2010), by assigning taxonomy with similarity levels of >80% for classes, >90% for families and >95% for genera (Tedersoo et al., 2015).

### 2.6 Data analyses

All tests were carried out in R 4.0.3 (R Core Team, 2021) with packages *ggplot2*, *indicspecies*, *miceco*, *multcomp*, *mvabund*, *phyloseq* and *vegan*, and significance level indicated as follows: * ≤ .05, ** ≤ .01, *** ≤ .001. ECM and SAP guilds were characterized based on taxonomy using FungalTraits (Põlme et al., 2020). When FungalTraits failed to assign a guild as a result of taxonomic uncertainty at the genus level, we treated these OTUs as ECM fungi when the closest MegaBLAST hit matched an ECM species hypothesis with >90% similarity and >90% coverage in UNITE (Abarenkov et al., 2024). We estimated ECM and SAP species richness by counting the number of OTUs shared between hosts in monodominant and mixed forests and visualized variation across forest type with species accumulation curves and Euler diagrams. We compared edaphic variables, enzyme activities, and percent relative abundance of ECM and SAP fungi between host species in monodominant and mixed stands, using generalized linear models (GLM), with a gamma distribution to accommodate continuous skewed variables. Pairwise comparisons were performed with Tukey's tests.

We visualized the relationships between ECM and SAP fungal community composition and biotic/abiotic predictors (host species and edaphic variables) with distance‐based redundancy analysis (dbRDA) by calculating a Bray–Curtis dissimilarity matrix, based on the relative abundance of OTUs detected in at least two samples, and stand (monodominant or mixed) as a condition. Significant predictors were selected based on *p*‐values and adjusted coefficients of determination (R2adj). Pairwise comparisons between co‐occurring hosts in mixed forests were performed using PerMANOVA. The proportion of variation in fungal community composition (Bray‐Curtis distances) explained by host independent of soil variables was explored with variation partitioning and visualized with Euler diagrams. We further explored how each of the selected predictors correlated with the relative abundance of fungal OTUs and genera in monodominant and mixed forests, by fitting multivariate GLMs using the *manyglm* function in *mvabund* (Wang et al., 2012): univariate analyses of deviance were performed with a negative binomial distribution for the 50 ECM and SAP OTUs with the highest relative abundance, as well as the 20 ECM and SAP genera or families with the highest relative abundance. A step‐down resampling procedure (999 permutations) was applied to account for multiple comparisons. Finally, we identified ECM and SAP fungal genera or families that were positively associated to a host species in monodominant and mixed forests based on fungal relative abundances, using point biserial correlation coefficients (De Cáceres & Legendre, 2009), with multiple testing accounted for using the Benjamin & Hochberg correction.

The correlation of enzyme activities with host species, edaphic variables, and biotic predictors (relative abundance of ECM and SAP fungi) was visualized with redundancy analysis, based on Euclidean distances and stand as a condition. Significant predictors were selected based on *p*‐values and R2adj, and pairwise comparisons between co‐occurring hosts in mixed forests were performed using PerMANOVA, as above. The proportion of variation in enzyme activities (Euclidian distances) explained by host, edaphic variables, and ECM/SAP relative abundances was examined with variation partitioning and visualized with Euler diagrams. We also tested whether each enzyme activity, as well as available N, net N mineralization and microbial biomass N correlated with the relative abundance of ECM fungi, SAP fungi, as well as the 20 ECM and SAP genera or families with the highest relative abundance, using GLM with a Gamma distribution. Models were run separately for each dependent variable and significance levels were adjusted with Bonferroni correction.

## 3 RESULTS

### 3.1 Fungal diversity overview

**FIGURE 2:**
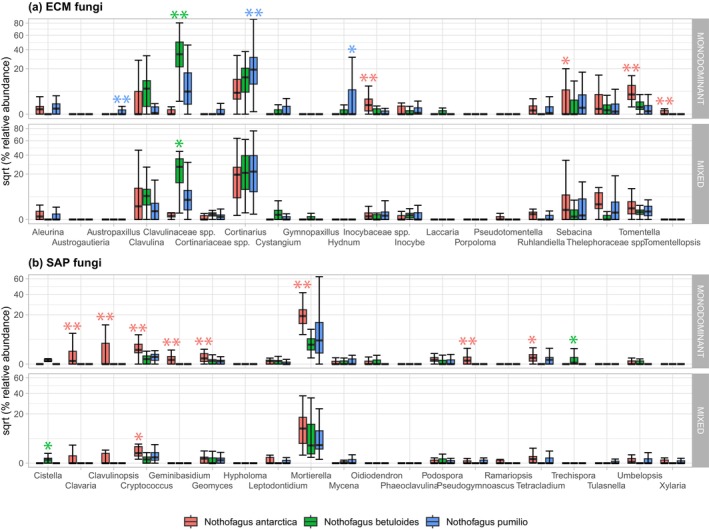
Relative abundance of (a) ectomycorrhizal (ECM) and (b) saprotrophic (SAP) fungal genera and families beneath each *Nothofagus* host, in monodominant and mixed forests. Square root transformation was applied to the graph to enhance visibility. Positive associations with a *Nothofagus* species based on point biserial correlation coefficients (Table S1) are indicated above the bars, with significance level as * ≤ .05, ** ≤ .01, adjusted using the Benjamin & Hochberg correction. Only the 20 ECM and SAP fungal groups with the highest relative abundance are shown here.

Two samples yielded fewer than 5000 sequences and were eliminated from the dataset, resulting in a total of 148 analyzed samples. The 3,695,961 sequences that passed quality filtering clustered into 1955 OTUs, including 749 ECM, 379 SAP and 54 pathogenic OTUs. A total of 803 OTUs (41%) were detected in only one sample, and species accumulation curves indicated that our sampling only captured a portion of the soil fungal diversity of the region (Figure S1). Observed ECM fungal richness was the highest beneath *N. pumilio* trees in mixed forests (Figure S2), while soil collected beneath monodominant *N. antarctica* trees had the highest number of SAP fungal OTUs. Genera with the highest relative abundance/frequency in our dataset were *Cortinarius* (395 ECM OTUs), *Mortierella* (66 SAP OTUs), *Clavulina* (36 ECM OTUs), *Inocybe* (27 ECM OTUs), *Sebacina* (24 ECM OTUs), and *Tomentella* (18 ECM OTUs), as well as ECM OTUs from the families Clavulinaceae (126 ECM OTUs), Inocybaceae (27 ECM OTUs), and Thelephoraceae (10 ECM OTUs, Figure 2). These OTUs could not be assigned to a fungal genus owing to the paucity of Patagonian fungi sequences, but matched closely to an ECM species hypothesis in UNITE.

### 3.2 Edaphic variables and enzymes activities across *Nothofagus* forests

In monodominant stands, soil pH and available P were significantly higher, and soil moisture significantly lower, beneath *N. antarctica* compared to the two other host species (Table 1). C:N ratio was significantly higher beneath *N. betuloides*, driven by a significantly higher total C. Surprisingly, soil beneath the evergreen *N. betuloides* had significantly higher available N, higher rates of net N mineralization and lower microbial biomass N than in deciduous stands of *N. antarctica* and *N. pumilio*, despite similar total N values. No significant differences in N and P availability were detected between hosts in mixed stands, although total C and C:N remained significantly higher beneath *N. betuloides*. Activities of the five C‐acquiring enzymes (AGLU, BGLU, GLUCU, CEL, and XIL) were significantly higher beneath *N. antarctica,* and fungal communities below this host species also had significantly higher relative abundance of SAP fungal OTUs per sample (Table 1). The N‐acquiring enzyme LEU was significantly higher beneath *N. pumilio*, and the N‐acquiring enzyme NAG was significantly higher beneath *N. betuloides*. Soil beneath both of these host trees had a higher relative abundance of ECM fungal OTUs than beneath *N. antarctica*. None of these differences remain significant in mixed stands, apart from the P‐acquiring enzyme PHOS whose activity was significantly higher beneath *N. betuloides* than *N. antarctica*.

**TABLE 1:** Mean and standard error of edaphic variables, enzyme activities and relative abundances of ectomycorrhizal (ECM) and saprotrophic (SAP) fungi variables across *Nothofagus* hosts in monodominant and mixed stands.

	Monodominant			Mixed		
	*N. antarctica*	*N. betuloides*	*N. pumilio*	*N. antarctica*	*N. betuloides*	*N. pumilio*
Soil moisture (%)	21.81 ± 6.24 ***a***	37.96 ± 12.03 ***b***	38.32 ± 14.81 ***b***	27.67 ± 9.70 ***a***	53.56 ± 21.60 ***c***	39.55 ± 15.95 ***b***
Soil pH	4.77 ± 0.31 ***c***	3.24 ± 0.44 ***a***	4.30 ± 0.48 ***b***	4.92 ± 0.49 ***b***	3.54 ± 0.69 ***a***	4.53 ± 0.88 ***b***
Total C (%)	11.56 ± 3.65 ***a***	24.66 ± 13.11 ***b***	13.94 ± 6.55 ***a***	13.26 ± 6.13 ***a***	27.03 ± 17.04 ***b***	17.16 ± 7.93 ***a***
Total N (%)	0.96 ± 0.34	0.83 ± 0.22	0.94 ± 0.42	1.40 ± 0.28 ***c***	0.76 ± 0.22 ***a***	0.96 ± 0.32 ***b***
C:N	14.55 ± 13.27 ***a***	29.08 ± 11.52 ***b***	17.23 ± 8.49 ***a***	10.43 ± 7.01 ***a***	34.06 ± 16.85 ***c***	19.74 ± 10.07 ***b***
Available N (ppm)	42.34 ± 14.49 ***a***	74.49 ± 28.66 ***b***	40.82 ± 13.11 ***a***	45.56 ± 17.33	52.06 ± 13.87	49.48 ± 13.16
Available P (ppm)	39.61 ± 24.69 ***c***	15.19 ± 9.21 ***a***	24.84 ± 17.41 ***b***	19.17 ± 12.23	21.34 ± 14.31	28.86 ± 22.49
Net N mineralization (μg N/g soil)	80.73 ± 8.63 ***b***	168.06 ± 20.61 ***c***	44.39 ± 12.52 ***a***	N/A	N/A	N/A
Microbial biomass N (μg N/g soil)	125.59 ± 19.00 ***b***	45.66 ± 7.86 ***a***	210.97 ± 29.32 ***c***	N/A	N/A	N/A
AGLU (nmol h^−1^ g^−1^)	7.27 ± 6.27 ***c***	1.28 ± 0.49 ***a***	3.44 ± 2.05 ***b***	4.19 ± 3.47	2.81 ± 1.39	3.72 ± 1.69
BGLU (nmol h^−1^ g^−1^)	30.84 ± 28.57 ***b***	17.71 ± 15.95 ***a***	23.78 ± 19.22 ***ab***	27.04 ± 22.64	35.88 ± 24.94	31.55 ± 18.70
GLUCU (nmol h^−1^ g^−1^)	37.44 ± 32.85 ***b***	14.34 ± 11.68 ***a***	12.65 ± 8.44 ***a***	27.73 ± 22.28	16.68 ± 17.24	21.92 ± 18.66
CEL (nmol h^−1^ g^−1^)	4.87 ± 3.56 ***c***	0.86 ± 0.81 ***a***	2.11 ± 2.37 ***b***	2.37 ± 2.29	2.94 ± 2.86	2.36 ± 1.42
XIL (nmol h^−1^ g^−1^)	7.18 ± 5.35 ***c***	1.77 ± 1.23 ***a***	3.11 ± 2.06 ***b***	4.12 ± 2.49	4.35 ± 3.84	3.80 ± 2.00
LEU (nmol h^−1^ g^−1^)	4.64 ± 3.21 ***a***	5.84 ± 3.29 ***a***	22.12 ± 11.06 ***b***	16.28 ± 9.03	10.66 ± 9.05	12.44 ± 6.33
NAG (nmol h^−1^ g^−1^)	176.49 ± 142.85 ***a***	513.96 ± 402.51 ***c***	320.83 ± 191.37 ***b***	326.87 ± 215.22	286.72 ± 181.51	309.07 ± 182.48
PHOS (nmol h^−1^ g^−1^)	94.82 ± 65.74	99.23 ± 36.10	73.55 ± 48.40	105.50 ± 54.80 ***a***	231.14 ± 152.02 ***b***	157.72 ± 106.96 ***ab***
ECM relative abundance (%)	37.36 ± 22.52 ***a***	67.99 ± 17.22 ***b***	54.24 ± 24.38 ***b***	45.53 ± 21.68	62.27 ± 16.89	56.11 ± 23.62
SAP relative abundance (%)	38.96 ± 17.68 ***c***	5.72 ± 3.60 ***a***	12.40 ± 14.74 ***b***	21.74 ± 11.90	10.09 ± 11.24	16.34 ± 24.38

*Note*: Tukey HSD post‐hoc tests with *p*‐values ≤.05 are indicated with bold letters, based on generalized linear models with a Gamma distribution.

Abbreviations: AGLU, alpha‐glucosidase; BGLU, beta‐glucosidase; CEL, cellobiohydrolase; GLUCU, beta‐glucuronidase; LEU, leucine aminopeptidase; NAG, N‐acetyl‐glucosaminidase; PHOS, acid phosphatase; XIL, beta‐xylosidase.

### 3.3 Predictors of fungal community composition

**FIGURE 3:**
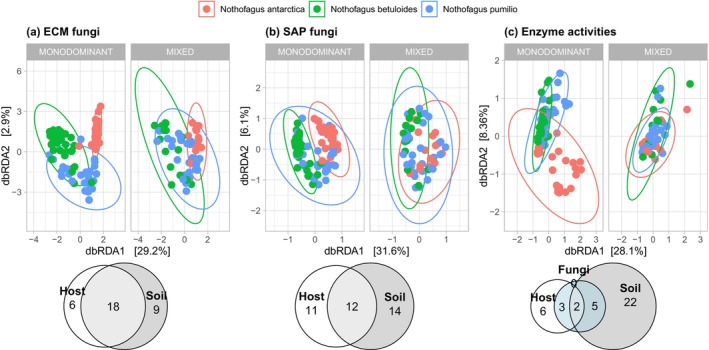
Distance‐based redundancy analysis of (a) ectomycorrhizal (ECM) and (b) saprotrophic (SAP) fungal community composition based on Bray‐Curtis distances and stand (monodominant or mixed) as a condition: Soil pH (*F* = 49.42***), host (*F* = 6.66***), soil moisture (*F* = 4.36***) and available N (*F* = 2.16**) were the best predictors of ECM fungal community structure, and 18% of the variation was explained by the interaction of host and soil variables, regardless of stand. Host (*F* = 22.88***), soil pH (*F* = 13.96***), and soil moisture (*F* = 10.93***) were the best predictors of SAP fungal community structure, and 12% of the variation was explained by the interaction of host and soil variables. Differences in fungal community composition were non‐significant between co‐occurring hosts in mixed forests. (c) Redundancy analysis of soil enzyme activities based on Euclidean distances, with samples colored by *Nothofagus* species: Soil moisture (39.62***), soil pH (*F* = 13.23***), host (*F* = 10.96***), and available P (*F* = 7.37***) were the best predictors of the variation in enzyme activities, with soil variables explaining 22% of the variation in enzyme activities.

Based on Bray‐Curtis distances, the best predictors of ECM fungal community composition were soil pH (*F* = 49.42***), host (*F* = 6.66***), soil moisture (*F* = 4.36***), and available N (*F* = 2.16**), while the best predictors of SAP fungal community composition were host (*F* = 22.88***), soil pH (*F* = 13.96***), and soil moisture (*F* = 10.93***) (Figure 3a,b). According to variation partitioning, variation in ECM and SAP fungal communities was explained primarily by edaphic variables (9% for ECM, 14% for SAP) and their interaction with host (18% for ECM, 12% for SAP, Figure 3a,b). Nevertheless, we did not find significant differences in fungal community composition between co‐occurring host species in mixed forests. Based on multivariate GML, in monodominant forests, host correlated significantly with eight ECM and 11 SAP fungal genera or families (Table 2), as well as 17 ECM and 32 SAP OTUs belonging to *Clavulina*, *Cortinarius*, and *Mortierella*, among others (Table S2). Except for some ECM Clavulinaceae spp., none of these correlations remained significant in mixed forests. Soil pH significantly correlated with the ECM genus *Aleurina* (specifically *A. argentina*) in both monodominant and mixed forests, while the ECM *Ruhlandiella*, Clavulinaceae spp., a *Sebacina* sp., and two SAP fungal genera correlated significantly with soil pH in monodominant forests (Table 2, Table S2). Additionally, soil moisture significantly correlated with *Mortierella*. Based on point biserial correlation coefficients, in monodominant stands, the ECM *Tomentella* and *Tomentellopsis*, as well as Inocybaceae spp. and Thelephoraceae spp. positively associated with *N. antarctica*, Clavulinaceae spp. with *N. betuloides*, and *Austropaxillus*, *Cortinarius* and *Hydnum* with *N. pumilio* (Figure 2a, Table S1). However, in mixed stands, only Clavulinaceae spp. associated positively with *N. betuloides*, while all other associations remained non‐significant. Regarding SAP fungi in monodominant forests, OTUs from 18 genera (including *Mortierella*) associated positively with *N. antarctica*, compared to only three genera with *N. betuloides*, and *Hymenocyphus* with *N. pumilio* (Figure 2b, Table S1). In mixed stands, only *Cistella* associated positively with *N. betuloides* and *Cryptococcus* with *N. antarctica*, while all other associations remained non‐significant.

**TABLE 2:** Correlations of fungal relative abundances with host, soil pH, and/or soil moisture in monodominant and mixed forests, based multivariate generalized linear models with a negative binomial distribution.

	Monodominant forests			Mixed forests		
	Host	Soil pH	Soil moisture	Host	Soil pH	Soil moisture
ECM fungi						
*Aleurina*	3.073	15.366**	2.716	1.087	17.999**	2.773
*Austropaxillus*	29.019***	0.200	0.230	0.747	0.048	2.693
*Clavulinaceae* spp.	22.861***	12.759*	0.432	9.400	7.796	3.984
*Cortinarius*	16.140**	0.035	6.614	0.302	1.465	10.500
*Cystangium*	21.693***	4.108	5.846	7.277	0.213	10.313
*Hydnum*	8.682*	4.288	0.151	4.187	0.051	4.444
*Inocybaceae* spp.	14.826**	1.369	2.803	1.119	1.699	0.219
*Laccaria*	16.561**	0.005	0.755	4.680	2.015	3.506
*Ruhlandiella*	4.585	14.874**	0.122	1.601	3.586	0.337
*Thelephoraceae* spp.	6.323	3.524	1.618	3.517	16.447**	12.479
*Tomentella*	30.677***	1.188	1.518	4.005	2.165	3.526
SAP fungi						
*Cistella*	3.861	0.309	0.276	12.504*	25.878**	0.000
*Clavaria*	17.483***	2.232	2.643	2.894	5.494	2.066
*Clavulinopsis*	17.841***	0.257	0.017	3.857	0.000	1.493
*Cryptococcus*	25.616***	5.412	4.177	9.710	9.009	0.000
*Geminibasidium*	28.931***	1.473	9.381	7.179	2.413	0.073
*Geomyces*	14.736**	2.579	1.483	0.117	4.277	3.668
*Mortierella*	36.505***	4.873	24.555*	1.571	8.747	1.265
*Pseudogymnoascus*	37.924***	11.682*	5.612	2.092	18.010**	11.619
*Ramariopsis*	9.180*	0.059	0.001	5.750	2.053	2.725
*Tetracladium*	15.865**	36.685***	0.001	6.209	16.582**	0.013
*Tulasnella*	9.838*	0.020	0.118	0.874	0.393	0.162
*Xylaria*	12.222**	0.006	1.006	9.628	7.229	27.400

*Note*: *F* values (univariate tests) are indicated with significance level as * ≤ .05, ** ≤ .01, *** ≤ .001 (in bold) adjusted using a step‐down resampling procedure (999 permutations). Only fungal genera and families showing a significant correlation are shown here.

### 3.4 Predictors of enzyme activities and N cycling across *Nothofagus* forests

Based on Euclidian distances, the best predictors of enzyme activities were soil moisture (39.62***), pH (*F* = 13.23***), host (*F* = 10.96***), and available P (*F* = 7.37***), while edaphic variables primarily explained the variation (22%) in enzymatic activities (Figure 3c). Despite the fact that ECM and SAP fungi were in general poor predictors of enzyme activities compared to edaphic variables, activities of the five C‐acquiring enzymes (AGLU, BGLU, GLUCU, CEL, and XIL) were significantly positively correlated with the relative abundance of SAP fungi and/or several SAP fungal genera, including *Mortierella* (Table 3). C‐acquiring enzymes were significantly negatively correlated with ECM fungi and/or *Cortinarius*, *Cystangium* and Clavulinaceae spp., while Inocybaceae spp. and Thelephoraceae spp. showed a significant positive correlation (Table 3). The N‐acquiring enzyme LEU was significantly positively correlated with *Aleurina* and negatively with Clavulinaceae spp., *Clavulinopsis* and *Geminibasidium*, while the P‐acquiring enzyme PHOS was significantly positively correlated with *Cistella*. Additionally, the relative abundance of Clavulinaceae spp. was significantly positively correlated with available N and net N mineralization, and negatively correlated with microbial biomass N.

**TABLE 3:** Correlations of fungal relative abundances with enzyme activities, available N, net N mineralization, and microbial biomass N, based on generalized linear models with a Gamma distribution.

	AGLU	BGLU	CEL	GLUCU	LEU	NAG	PHOS	XIL	Available N	Microbial biomass N	Net N mineralization
ECM fungi	−5.314***	−3.267	−5.401***	−5.221***	−2.039	0.714	−0.913	−4.791***	4.056**	−2.366	3.209
*Aleurina*	1.556	0.881	1.363	2.302	3.465*	0.771	−1.794	0.467	−1.608	2.903	−3.149
*Clavulinaceae* spp.	−7.540***	−2.526	−3.862**	−4.233**	−3.919**	2.012	1.495	−3.509*	7.382***	−4.323**	4.962***
*Cortinarius*	−1.764	−1.159	−3.802**	−2.443	1.224	−1.042	−0.836	−3.195	−1.456	1.155	−1.134
*Cystangium*	−2.924	−3.237	−3.677*	−4.416***	−0.895	−1.479	−1.201	−3.219	−0.192	0.474	−0.704
*Hydnum*	−1.945	−1.745	−2.926	−2.536	−0.208	−0.246	−1.683	−3.351*	−0.545	0.187	0.026
*Inocybaceae* spp.	4.494***	2.758	3.465*	1.648	−1.065	−1.685	1.161	4.007**	−3.391*	−0.086	−1.037
*Thelephoraceae* spp.	2.896	1.845	2.640	3.352*	0.796	0.671	−0.368	2.205	−0.597	−0.385	−0.146
*Tomentella*	3.650*	1.473	2.018	0.678	−0.951	−1.041	0.151	3.286	−1.502	−0.845	−0.298
SAP fungi	6.748***	2.749	6.383***	7.093***	0.529	−0.701	1.412	6.301***	−2.567	0.800	−1.978
*Cistella*	0.286	1.695	1.309	0.324	−0.476	0.970	4.512***	1.604	3.389*	−1.337	2.192
*Clavulinopsis*	0.656	−2.002	0.643	2.331	−3.948**	−2.999	−2.016	0.936	−1.497	0.182	−0.751
*Cryptococcus*	3.550*	0.292	3.444*	6.112***	−2.598	−0.646	−1.576	2.964	−1.777	0.715	−0.917
*Geminibasidium*	0.576	−1.492	0.740	1.614	−3.826**	−1.460	−1.194	0.686	−1.879	0.364	−0.378
*Geomyces*	3.981**	1.987	2.143	2.094	−0.861	−0.069	0.306	2.651	−2.184	−0.143	−0.565
*Mortierella*	6.350***	3.326*	6.375***	5.331***	2.053	0.283	2.232	6.029***	−1.592	0.898	−2.206
*Pseudogymnoascus*	3.494*	1.932	3.253	2.098	−1.169	−1.732	−0.383	3.079	−1.814	0.082	−0.525
*Tetracladium*	4.944***	2.875	5.078***	4.309***	1.918	0.554	0.139	3.344*	−2.722	0.462	−1.005
*Trechispora*	−2.176	−1.300	−1.616	−1.436	−3.096	−0.838	−0.374	−1.570	4.438***	−0.865	1.188

*Note*: *t*‐Values are indicated with significance level as * ≤ .05, ** ≤ .01, *** ≤ .001, adjusted using Bonferroni corrections. Only fungal genera and families showing a significant correlation are shown here.

Abbreviations: AGLU, alpha‐glucosidase; BGLU, beta‐glucosidase; CEL, cellobiohydrolase; ECM, ectomycorrhizal; GLUCU, beta‐glucuronidase; LEU, leucine aminopeptidase; NAG, N‐acetyl‐glucosaminidase; PHOS, acid phosphatase; SAP, saprotrophic; XIL, beta‐xylosidase.

## 4 DISCUSSION

### 4.1 Inorganic N economy in evergreen *Nothofagus betuloides* forests

**FIGURE 4:**
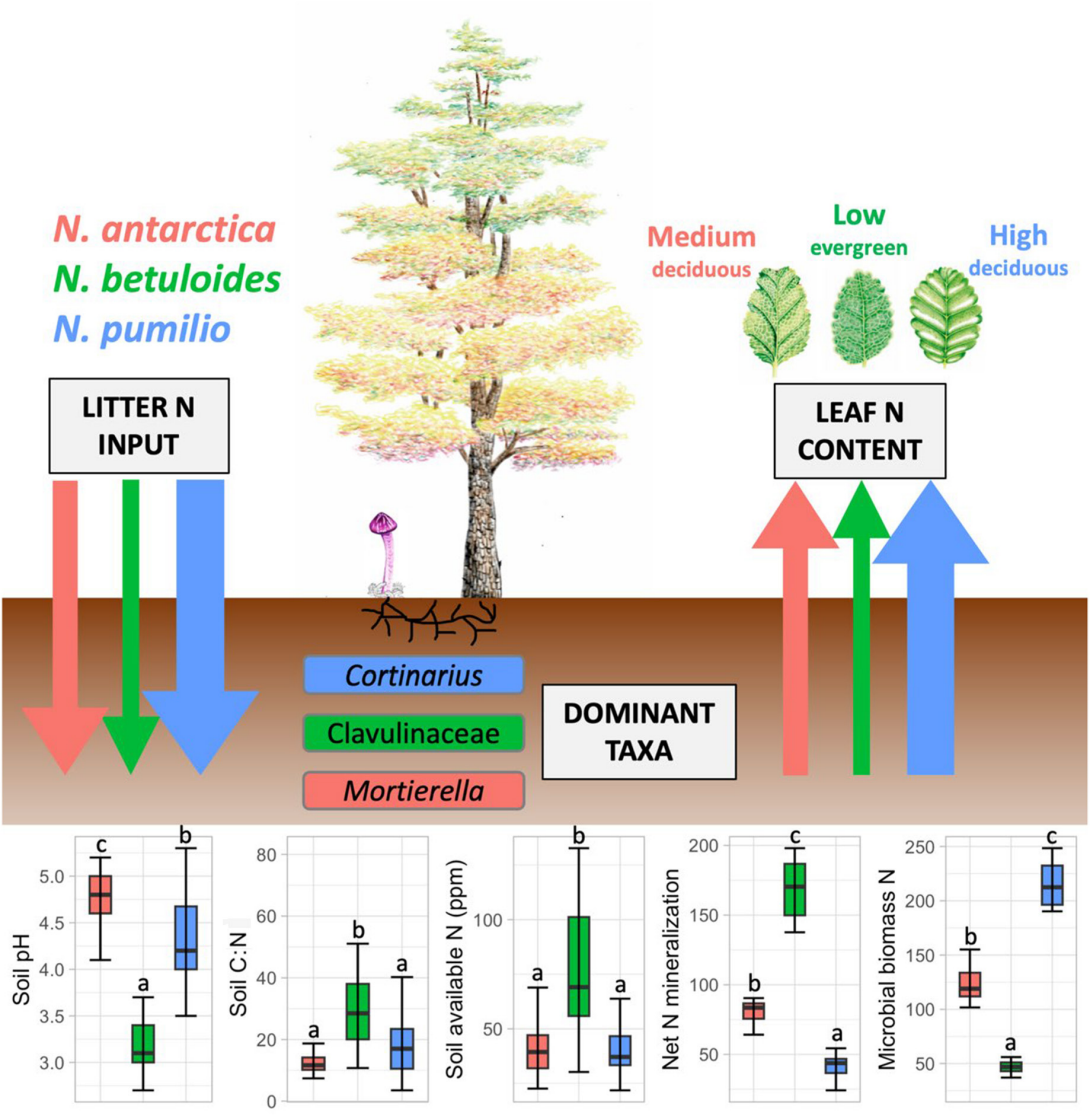
The nitrogen (N) economy in monodominant *Nothofagus* forests of Tierra del Fuego: Arrows represents hypothetical N fluxes color‐coded by *Nothofagus* species, based on N litter input and N leaf content according to previous studies (Diehl et al., 2008; Moretto & Martínez Pastur, 2014; Romanyà et al., 2005) Edaphic variables are represented as boxplots, with Tukey HSD post‐hoc tests (*p*‐values ≤.05) indicated with bold letters, based on generalized linear models with a Gamma distribution, as in Table 1. Despite a lower N leaf content and litter N input, N availability was significantly higher in evergreen *N. betuloides* forests (in green), suggesting a tight *inorganic* N cycle with higher relative abundance of ECM taxa (Clavulinaceae) adapted to low soil pH and poor drainage. In comparison, N availability was significantly lower in deciduous *N. pumilio* forests (in blue), despite higher N leaf content and litter N input; significantly higher N microbial biomass and relative abundance of ECM fungi with SOM decay abilities (*Cortinarius*) suggest an *organic* N economy where N is primarily stored in ECM mycelial biomass. Soils in deciduous *N. antarctica* forests (in red) showed intermediate levels of N availability and a higher relative abundance of saprotrophic fungi (*Mortierella*), likely promoted by understory grassland vegetation. Illustrations © PameFagus (Pamela Ciudad Martin).

Contrary to our first hypothesis that N cycling would be the slowest beneath *N. betuloides*, we found that available N and net N mineralization were significantly higher in evergreen monodominant forests than beneath deciduous *N. antarctica* and *N. pumilio* (Table 1, Figure 4). Staelens et al. (2011) observed similar patterns further north in the temperate rainforests of Chile, where they measured higher N mineralization rates in evergreen *N. dombeyi* forests than in nearby deciduous forests. Soils in those temperate rainforests originate from volcanic ashes with varying drainage capacity (Godoy et al., 2013), while in subantarctic forests of Tierra del Fuego, soils are of glacial origin. In both cases, high rainfall (up to 4000 mm/year) in these coastal regions can cause temporary waterlogging (Piper et al., 2008; Romanyà et al., 2005), but minimal hydrological loss of inorganic N has been measured (Oyarzún et al., 2004; Perakis & Hedin, 2001). Together, these results suggest a tight N cycle and mechanisms to limit N losses in waterlogged environments: Processes such as dissimilatory nitrate reduction to ammonium, coupled with the rapid N assimilation and aboveground transfer mediated by soil microbes prevent inorganic N leaching and contribute to long‐term N retention in evergreen *Nothofagus* forests that receive high rainfall (Huygens et al., 2008; Perakis & Hedin, 2001). Similarly, in the perhumid temperate rainforests of the Pacific Northwest, high rainfall and N fertility conditions favored endemic ECM fungi adapted to high inorganic N (McPolin et al., 2024). Here, we measured net N mineralization under standardized laboratory conditions and further field measurements are needed to account for the spatial and temporal heterogeneity of Patagonian soils, as well as site‐specific effects of climate and drainage on N cycling. It would also be interesting to further compare ECM diversity patterns and functions across temperate rainforests in correlation with soil fertility and rainfall at the global scale.

The positive correlation of ECM Clavulinaceae with available N and net N mineralization (Table 3) indicate that this group likely plays a functional role in N cycling in acidic soils. Members of the Clavulinaceae form ECM associations with a wide range of plant families in temperate regions, including *Nothofagus* (Orlovich et al., 2013; Uehling et al., 2012). These ECM taxa are typically abundant in acidic forest soils (Argüelles‐Moyao et al., 2017; Truong et al., 2019) and evidence suggests that Clavulinaceae are sensitive to increases in soil pH (Kluber et al., 2012). This is in line with our findings that showed a significant correlation of ECM Clavulinaceae with both host and soil pH in monodominant forests (Table 2, Table S1). ECM Clavulinaceae were also positively associated with *N. betuloides*, consistent with a deeper organic horizon, significantly lower soil pH, and higher total C and C:N ratio than in deciduous forests (Figure 2, Table 1, Table S2). Although the N‐acquisition strategies of ECM Clavulinaceae from Patagonia are not well studied, *Clavulina* species from other parts of the world are known for their capacity to acquire inorganic N, particularly ammonium (Khokon et al., 2023). Given the assumption that plant dynamically allocate C to the ECM fungal species that more effectively transfer N, the high metabolic costs of mining N from SOM become disadvantageous to plants and their fungal partners in high inorganic N conditions (Van Der Linde et al., 2018). As a consequence, a species turnover is often observed from ECM species with SOM decay abilities, such as *Cortinarius*, to ECM fungi that form short‐distance exploration mycorrhizae and readily utilize inorganic N, such as *Clavulina* (Defrenne et al., 2019; Kranabetter et al., 2015; Pellitier & Zak, 2021) as N availability increases.

### 4.2 Organic nitrogen economy in deciduous *Nothofagus pumilio* forests

Despite higher leaf N content and litter input of *N. pumilio* (Diehl et al., 2008; Romanyà et al., 2005), soils in these deciduous forests had significantly lower available N and net N mineralization compared to evergreen *N. betuloides* forests (Table 1, Figure 4). This surprisingly low soil fertility suggests an N economy dominated by organic N, where most N is stored in ECM mycelial biomass, as illustrated by the significantly higher microbial biomass N of *N pumilio*. Similar patterns have been repeatedly described in boreal forests dominated by evergreen conifers, where N limitation favors ECM fungi that possess enzymatic pathways to decompose complex organic N sources, such as *Cortinarius* (Castaño et al., 2023; Lindahl et al., 2021). *Cortinarius* is hyper‐diverse in Patagonian forests (Truong et al., 2017) and was the most species‐rich and abundant fungal genus in our dataset. *Cortinarius* was also positively associated with *N. pumilio* in monodominant forests (Figure 2). Nonetheless, although the relative abundance of *Cortinarius* negatively correlated with available N and net N mineralization (as hypothesis two predicted), this correlation was non‐significant and may reflect variations in nutrient acquisition strategies between different *Cortinarius* species. The role of peroxidase fungal enzymes in Nothofagaceae forests needs further investigation, but our results suggest that N cycling in *N. pumilio* deciduous forests is strikingly similar to patterns observed in evergreen conifers from the boreal zone.

AM and non‐mycorrhizal (NM) plant species rely on other microorganisms for N mineralization and are unable to access nutrients bound in SOM (Bunn et al., 2019). The low N availability in *N. pumilio* forests may therefore promote positive feedbacks and provide exclusive access to organic soil N to ECM *Nothofagus* trees (Bennett & Klironomos, 2019; Castaño et al., 2023). This may explain why cold‐tolerant trees and shrubs (e.g., species of *Drimys* (AM), *Embothrium* (NM) or *Maytenus* (AM)), are mostly absent from *N. pumilio* subantarctic forests in southern Patagonia compared to temperate rainforests further north (Marín et al., 2018). On the other hand, AM trees, such as *Drimys winteri*, *Maytenus magellanica*, or *Pilgerodendron uvifera*, occur at low frequency on the more N‐rich soils beneath *N. betuloides* (Promis et al., 2008). Contrary to northern hemisphere forests, where AM trees tend to displace ECM trees in high fertility soils (Mao et al., 2019), these AM tree species never become dominant in subantarctic forests of Tierra del Fuego. However, the primers we used to generate ITS1 amplicons are not optimal for detecting AM fungi (Lekberg et al., 2018) and we therefore avoid making inferences about AM associations here. Additionally, some Ericaceae species are known to occur in low abundance in *N. pumilio* understory, such as *Empetrum rubrum* and *Gaultheria mucronata* (Mestre et al., 2017; Rosas et al., 2019). Ericoid mycorrhizal fungi possess extensive capabilities to degrade organic compounds and can strengthen the impact of ECM fungi on N availability, while competing with saprotrophic fungi for recalcitrant organic substrates (Ward et al., 2022). These interactions require further attention to fully understand N cycling in *Nothofagus* forests.

### 4.3 Dominance of saprotrophic fungi in *Nothofagus antarctica* forest soils

Contrary to the two other *Nothofagus* species, soil beneath *N. antarctica* harbored a higher richness and abundance of SAP fungi (Table 1, Figure S1), with 18 genera, including *Mortierella*, that were positively associated with *N. antarctica* in monodominant stands (Figure 2, Table S2). Litter decomposition rates increased in grasslands that spontaneously grow under the *N. antarctica* canopy (Bahamonde et al., 2012). Native guanacos naturally graze these forests, bringing additional inputs of organic materials and nutrients (Peri et al., 2016). These processes are likely to generate large N stocks and lower C:N ratio in favor of SAP fungi (Castaño et al., 2023). Accordingly, we measured higher activities of the five C‐acquiring enzymes in soil beneath *N. antarctica* that positively correlated with the relative abundances of several SAP genera, including *Mortierella* (Table 3). Increase in soil nutrients, especially phosphorus, often correlates with increase in SAP fungal abundances (Khalid et al., 2021; Kyaschenko et al., 2017), as illustrated by higher available P measured in *N. antarctica* soils (Table 1).

Despite the generally lower abundance of ECM fungi in *N. antarctica* soils, some ECM lineages, i.e. *Tomentella*, *Tomentellopsis*, as well as ECM OTUs from Inocybaceae and Thelephoraceae, positively associated with *N. antarctica* in monodominant stands (Figure 2, Table S1). Competition with understory grassland plants can negatively affect the establishment of *N. antarctica* seedlings (Bahamonde et al., 2018); *Tomentella* and *Inocybe* species typically associate with seedlings (Kuhar et al., 2016) and may therefore play a role in the recruitment of *N. antarctica*.

### 4.4 Environmental filtering of the soil mycobiota in *Nothofagus* forests

Most of the differences in fungal community composition, nutrient availability and enzyme activities between *Nothofagus* host species did not persist in mixed forests (Table 1, Figure 3) These results are consistent with our hypothesis that edaphic variables are strong underlying factors affecting host distribution and N cycling in southern Patagonia. Soil pH was the strongest predictor of fungal community composition in *Nothofagus* forests (Figure 3, Table 2), as previous suggested (Longo et al., 2011; Truong et al., 2019), and correlated significantly with the ECM fungal groups *Aleurina*, Clavulinaceae spp., *Ruhlandiella*, and *Sebacina* (Table 3, Table S2). Litter quality can affect nutrient cycling and microbial activities in soils (Bennett & Klironomos, 2019), in line with the combined effect of host and soil pH on fungal communities (Table 2). By shedding recalcitrant litter, evergreen *N. betuloides* trees further acidify the soil, magnifying the effect of pH on soil microbes (Tedersoo & Bahram, 2019). Soil pH can also affect the mobility and availability of nutrients in soils, by altering the solubility of minerals and the uptake of nutrients by plant roots, therefore playing a pivotal role for SOM recycling and plant nutrition in ECM forests (Barrow & Hartemink, 2023; Husson, 2013).

Apart from ECM Clavulinaceae, most fungal taxa were not associated with a particular host species in mixed forests (Figure 2, Tables S1 and S2). Host preference is generally low at the generic level (Lofgren et al., 2018), including in *Nothofagus* (Nouhra et al., 2013). Nevertheless, our correlations are based on common fungal taxa, while host tree identity can have greater effects on rare symbiont species (van Galen et al., 2023). Because our dataset likely underestimated the soil fungal diversity of the region (Figure S1), we purposefully avoided making assumptions about alpha‐diversity patterns between *Nothofagus* host species. Further studies looking more specifically at fungi colonizing the roots of co‐occurring *Nothofagus* species with different leaf phenology are needed to further explore host preference in these forests.

More than 50% of the variation in ECM and SAP fungal communities remained unexplained by host and/or the edaphic variables measured (Figure 3), suggesting that other co‐occurring factors contribute to fungal community assembly in *Nothofagus* soils. Sharp climatic gradients shape the vegetation of Tierra del Fuego (Figure 1), with *N. pumilio* receiving less precipitation and experiencing greater temperature variation than *N. betuloides*, while *N. antarctica* is exposed to severe precipitation and temperature fluctuations, leading to temporal drought stress (Frangi et al., 2005; Musotto et al., 2017). Soil moisture was the best predictor of enzyme activities (Figure 3), illustrating the putative effect of water regime on microbial functions in soils with varying drainage capacity. ECM and soil fungal communities are also likely to vary between seasons (Beidler et al., 2023), particularly regarding litter input and decomposition (Vořiškova et al., 2014). Repeated sampling effort is therefore needed to account for the temporal dynamics of soil fungal communities in *Nothofagus* forests. Such information is critical to conserve these forests in a changing climate, but knowledge gaps in the southern hemisphere currently limit our ability to predict ECM fungal responses to climate change (Bennett & Classen, 2020).

## 5 CONCLUSIONS

Our findings illustrate the diversity of N acquisition strategies of ECM fungi in *Nothofagus* forests. Although the patterns we observed are not causative relationships, they are congruent with an organic N economy in deciduous *N. pumilio* forests, similarly to boreal forests dominated by evergreen conifers. In contrast, we found evidence of an inorganic N economy in evergreen *N. betuloides* forests, putatively linked to soil acidity and poor drainage, that showed similitudes with high fertility soils of coniferous temperate rainforests of the Pacific Northwest. In deciduous *N. antarctica* forests, grassy understory vegetation likely promoted SAP fungi that correlated with higher activities of C‐degrading enzymes. Our results illustrate the strong underlying effects of soil and climate on *Nothofagus* distribution and N cycling in southern Patagonia, regardless of leaf phenology. The range of soil fertility of these ECM angiosperms illustrate the adaptability of ECM fungi to a variety of conditions that do not always reflect northern hemisphere paradigms, where conifers are the dominant ECM hosts. Prediction modeling studies indicate that the potential habitat of *Nothofagus* species is likely to decrease overall in response to climate change (Mathias et al., 2023). This study lays the foundation for future research on the role of ECM fungi for nutrient cycling in subantarctic forests and their adaptability in a changing climate.

## AUTHOR CONTRIBUTIONS

**Camille Truong:** Conceptualization (lead); data curation (lead); formal analysis (lead); funding acquisition (lead); investigation (lead); project administration (lead); writing – original draft (lead); writing – review and editing (lead). **Luciano A. Gabbarini:** Conceptualization (supporting); data curation (supporting); formal analysis (supporting); funding acquisition (supporting); investigation (equal); project administration (supporting); writing – review and editing (equal). **Alicia Moretto:** Conceptualization (supporting); data curation (supporting); formal analysis (supporting); funding acquisition (supporting); investigation (equal); project administration (supporting); writing – review and editing (equal). **Julio M. Escobar:** Conceptualization (supporting); investigation (equal); writing – review and editing (equal). **Matthew E. Smith:** Conceptualization (lead); data curation (supporting); formal analysis (supporting); funding acquisition (lead); investigation (supporting); project administration (lead); writing – original draft (supporting); writing – review and editing (equal).

## FUNDING INFORMATION

This work was supported by the US National Science Foundation (DEB1354802 to MES), the National Scientific and Technical Research Council of Argentina (CONICET) and the Swiss National Science Foundation (Advanced Postdoc Mobility fellowship P300P3_158523 to CT).

## CONFLICT OF INTEREST STATEMENT

The authors have no conflict of interest to declare.

## DECLARATION

Our study brings together authors from a number of different countries, including scientists based in the country (LAG) and the region (JME and AM) where the study was carried out. All authors were engaged early on with the research and study design to ensure that the diverse sets of perspectives they represent was considered from the onset. Whenever relevant, literature published by scientists from the region was cited and efforts were made to consider relevant work published in the local language. We also provided a second abstract in Spanish to stimulate the diffusion of our work in the region.

## Supporting information


Data S1.

## Data Availability

Raw sequence and meta‐data were deposited at NCBI's Sequence Read Archive, Bioproject PRJNA476118. Samples and data from the PUM plots correspond to the three south‐exposed lowland plots in Truong et al. (2019). In house custom scripts are available at https://github.com/camillethuyentruong/Illumina_paired_end.
