# Supplementary material for: Ectomycorrhizal fungi and the nitrogen economy of Nothofagus in southern Patagonia
Source: Ecol Evol. 2024 Sep 29;14(10):e70299. doi: 10.1002/ece3.70299 (PMC11439510; doi:10.1002/ece3.70299)
Supplement: Supplementary file 1 — Data S1. [file ECE3-14-e70299-s001.docx]

**Supporting information:**

**Ectomycorrhizal fungi and the nitrogen economy of *Nothofagus* in southern Patagonia**

**
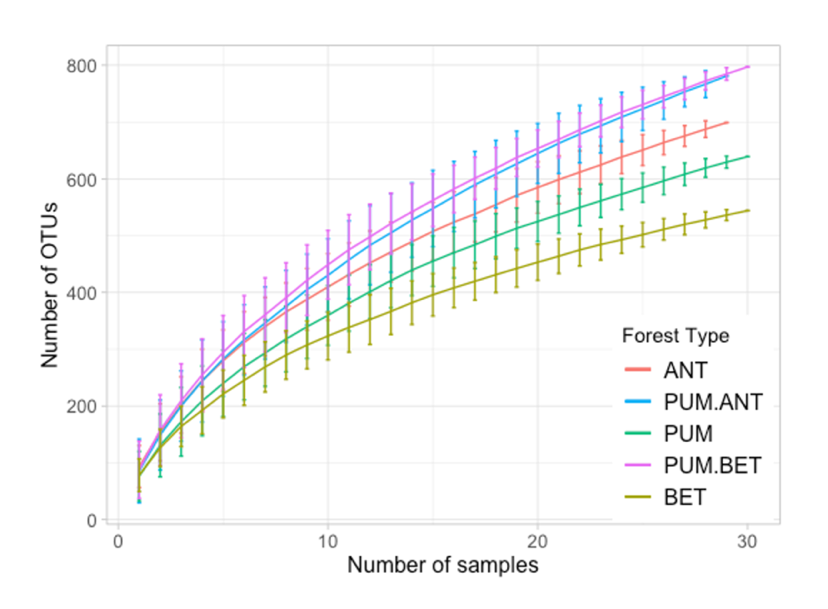
**

**Figure S1** Species accumulation curves representing the number of OTUs recovered with increasing sample effort in each forest type. ANT=N. antarctica, PUM.ANT=mixed pumilio-antarctica, PUM=N. pumilio, PUM.BET= mixed pumilio-betuloides, BET=N. betuloides.

**
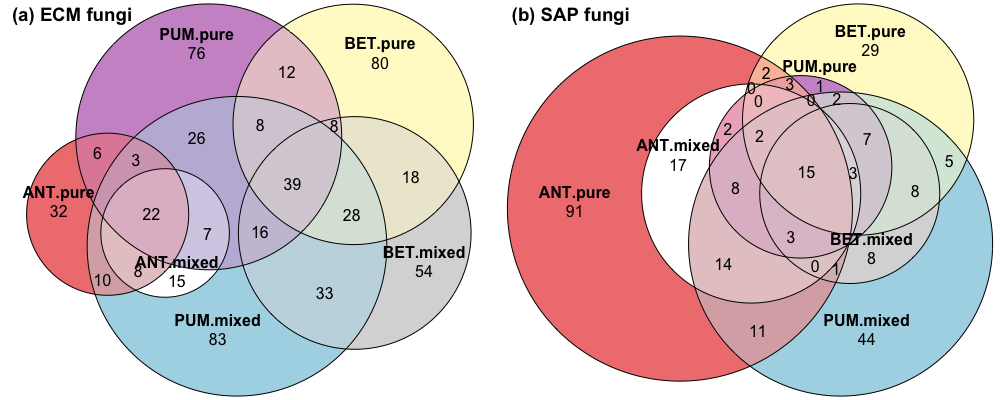
**

**Figure S2** Number of (a) ectomycorrhizal (ECM) and (b) saprotrophic (SAP) fungal OTUs shared between hosts in monodominant and mixed forests: ANT=*N. antarctica*, PUM=*N. pumilio*, BET=*N. betuloides*, pure=monodominant forests, mixed=mixed forests.

**Table S1** Positive associations of ectomycorrhizal (ECM) and saprotrophic (SAP) fungal genera and families with *Nothofagus* host species in monodominant and mixed forests, based on point biserial correlation coefficients (r_pb_) with significance level as *≤0.05, **≤0.01, adjusted using

the Benjamini & Hochberg correction.

|  | r_pb_ | Host species |
| --- | --- | --- |
| ECM FUNGI (MONODOMINANT FORESTS) | | |
| *Austropaxillus* | 0.377** | *N. pumilio* |
| *Clavulinaceae spp.* | 0.713** | *N. betuloides* |
| *Cortinarius* | 0.409** | *N. pumilio* |
| *Hydnum* | 0.298* | *N. pumilio* |
| *Inocybaceae spp.* | 0.437** | *N. antarctica* |
| *Thelephoraceae spp.* | 0.333* | *N. antarctica* |
| *Tomentella* | 0.426** | *N. antarctica* |
| *Tomentellopsis* | 0.309** | *N. antarctica* |
| SAP FUNGI (MONODOMINANT FORESTS) | | |
| *Aspergillus* | 0.256** | *N. antarctica* |
| *Auricularia* | 0.312* | *N. antarctica* |
| *Cladosporium* | 0.479** | *N. antarctica* |
| *Clavaria* | 0.352** | *N. antarctica* |
| *Clavulinopsis* | 0.392** | *N. antarctica* |
| *Cryptococcus* | 0.595** | *N. antarctica* |
| *Entoloma* | 0.477** | *N. betuloides* |
| *Geminibasidium* | 0.340** | *N. antarctica* |
| *Geomyces* | 0.292** | *N. antarctica* |
| *Glarea* | 0.282** | *N. antarctica* |
| *Hymenoscyphus* | 0.463** | *N. pumilio* |
| *Leohumicola* | 0.295* | *N. antarctica* |
| *Mortierella* | 0.541** | *N. antarctica* |
| *Mrakia* | 0.408** | *N. antarctica* |
| *Penicillium* | 0.310* | *N. antarctica* |
| *Peziza* | 0.405** | *N. antarctica* |
| *Psathyrella* | 0.290* | *N. antarctica* |
| *Pseudogymnoascus* | 0.394** | *N. antarctica* |
| *Rhodotorula* | 0.461** | *N. antarctica* |
| *Tetracladium* | 0.301* | *N. antarctica* |
| *Torrendiella* | 0.343* | *N. betuloides* |
| *Trechispora* | 0.314* | *N. betuloides* |
| ECM FUNGI (MIXED FORESTS) | | |
| *Clavulinaceae spp.* | 0.593* | *N. betuloides* |
| SAP FUNGI (MIXED FORESTS) | | |
| *Cistella* | 0.445* | *N. betuloides* |
| *Cryptococcus* | 0.423* | *N. antarctica* |

**Table S2** Correlations of fungal relative abundances with host species, soil pH and/or soil moisture in monodominant and mixed forests, based multivariate generalized linear models with a negative binomial distribution. F values (univariate tests) are indicated with significance level as *≤0.05, **≤0.01, ***≤0.001 (in bold) adjusted using a step-down resampling procedure (999 permutations). Only ectomycorrhizal (ECM) and saprotrophic (SAP) fungal OTUs showing a significant correlation are shown here.

|  | MONODOMINANT FORESTS | | |  | MIXED FORESTS | | |
| --- | --- | --- | --- | --- | --- | --- | --- |
|  | Host | Soil  pH | Soil moisture |  | Host | Soil  pH | Soil moisture |
| ECM FUNGI |  |  |  |  |  |  |  |
| *Aleurina argentina* | 3.073 | **15.366*** | 2.716 |  | 1.087 | **17.999*** | 2.773 |
| *Clavulina sp.* | **28.661***** | 3.074 | 0.402 |  | 7.926 | **26.697**** | 5.747 |
| *Clavulina sp.* | **50.894***** | 1.990 | 1.311 |  | 3.565 | 8.111 | 1.446 |
| *Clavulina sp.* | **18.210**** | 3.895 | 11.325 |  | **21.289**** | 7.654 | 5.820 |
| *Clavulinaceae sp.* | **27.417***** | 10.256 | 0.267 |  | **15.642*** | **42.506***** | 0.375 |
| *Clavulinaceae sp.* | **29.713***** | 12.967 | 0.390 |  | **19.427**** | **28.410**** | -0.078 |
| *Clavulinaceae sp.* | **11.845*** | 0.012 | 0.250 |  | 11.547 | **27.269**** | 2.778 |
| *Clavulinaceae sp.* | **23.941***** | 0.859 | 0.535 |  | **17.687**** | 15.562 | 6.165 |
| *Clavulinaceae sp.* | **36.794***** | 1.668 | 3.343 |  | **14.371*** | 12.147 | 5.873 |
| *Clavulinaceae sp.* | **21.915**** | 4.871 | 0.384 |  | 0.667 | 1.392 | 0.941 |
| *Clavulinaceae sp.* | 3.284 | 0.169 | 0.346 |  | **12.797*** | 1.269 | 2.936 |
| *Cortinarius dulciolens* | 9.287 | 0.005 | 1.521 |  | 8.515 | **20.748**** | 1.942 |
| *Cortinarius lubricanescens* | **19.040**** | 1.059 | 0.002 |  | 7.408 | 0.024 | 0.342 |
| *Cortinarius sp.* | **14.086*** | 0.063 | 0.029 |  | 9.495 | 2.513 | 7.624 |
| *Hydnum sp.* | **22.333**** | 7.438 | 4.036 |  | 1.380 | 15.843 | 0.153 |
| *Hydnum sp.* | **14.647**** | 0.888 | 0.050 |  | 1.380 | 2.041 | 0.244 |
| *Sebacina sp.* | **14.091*** | **19.023**** | 1.060 |  | 0.626 | 0.070 | 0.079 |
| *Thelephoraceae sp.* | 0.467 | 9.356 | 1.600 |  | 1.829 | **24.547**** | 7.293 |
| *Thelephoraceae sp.* | **19.519**** | 1.300 | 3.555 |  | 9.169 | 2.918 | 3.505 |
| *Tomentella galzinii* | **51.799***** | 1.585 | 0.023 |  | 5.898 | 0.527 | 2.663 |
| *Tomentella sp.* | **69.447***** | 4.545 | 4.917 |  | 3.624 | 0.883 | 7.970 |
|  |  |  |  |  |  |  |  |
| SAP FUNGI |  |  |  |  |  |  |  |
| *Aspergillus ruber* | **14.898**** | 0.505 | 0.228 |  | 0.000 | 0.000 | 0.000 |
| *Byssonectria fusispora* | **10.556*** | 2.218 | 2.792 |  | 0.000 | 0.000 | 0.000 |
| *Cistella sp.* | **41.817***** | 5.880 | 3.873 |  | 12.504 | **25.878**** | 2.066 |
| *Clavaria sp.* | **48.084***** | 1.898 | 3.277 |  | 4.851 | 7.071 | 3.138 |
| *Clavaria sp.* | **26.712***** | 1.461 | 0.009 |  | 2.967 | 0.466 | 2.195 |
| *Clavaria sp.* | **17.491**** | 0.139 | 0.001 |  | 0.000 | 0.000 | 0.000 |
| *Clavulinopsis luteoalba* | **17.934**** | 0.114 | 0.117 |  | 5.380 | 10.050 | 6.319 |
| *Cryptococcus sp.* | **44.837***** | 0.129 | 1.525 |  | 0.000 | 0.000 | 0.000 |
| *Cryptococcus terreus* | **44.746***** | 0.223 | 0.916 |  | 0.000 | 0.000 | 0.000 |
| *Cryptococcus terricola* | **20.420***** | 5.164 | 3.629 |  | 9.424 | 8.681 | 0.054 |
| *Geminibasidium sp.* | **28.931***** | 1.473 | 9.381 |  | 7.179 | 2.413 | 1.265 |
| *Geomyces asperulatus* | **78.360***** | 9.095 | 0.045 |  | 9.585 | 7.076 | 0.783 |
| *Hymenoscyphus sp.* | **43.514***** | 0.073 | 3.355 |  | 0.171 | 8.516 | 1.126 |
| *Mortierella alpina* | **54.718***** | 0.571 | 0.107 |  | 11.293 | 4.052 | 1.577 |
| *Mortierella bainieri* | **9.604*** | 9.321 | 4.683 |  | 1.380 | 0.513 | 0.872 |
| *Mortierella fimbricystis* | **26.201***** | **22.987**** | 0.000 |  | 1.345 | **26.796**** | 1.638 |
| *Mortierella humilis* | **33.049***** | 0.000 | 4.895 |  | 4.742 | 5.836 | 9.869 |
| *Mortierella hyalina* | **29.706***** | 5.740 | 1.160 |  | 4.622 | 5.260 | 1.412 |
| *Mortierella parvispora* | **12.534*** | 0.051 | 0.884 |  | 2.601 | 6.053 | 1.251 |
| *Mortierella pseudozygospora* | **20.090***** | **19.029*** | 1.053 |  | 10.351 | 6.700 | 4.507 |
| *Mortierella sp.* | **26.073***** | 1.538 | 2.770 |  | 2.944 | 5.847 | 1.672 |
| *Mortierella sp.* | **10.184*** | **14.700*** | 2.364 |  | 4.754 | 0.000 | 1.598 |
| *Mortierella turficola* | **85.620***** | 11.960 | 0.036 |  | 2.815 | 0.020 | 2.014 |
| *Mycena sp.* | **22.642***** | 0.056 | 0.432 |  | 4.379 | 4.558 | 9.328 |
| *Peziza howsei* | **37.051***** | 3.374 | 3.276 |  | 6.112 | 0.141 | 0.067 |
| *Pseudogymnoascus verrucosus* | **20.564***** | 13.109 | 0.345 |  | 1.380 | **16.733*** | 0.008 |
| *Ramariopsis flavescens* | **9.611*** | 0.027 | 3.377 |  | 12.916 | 1.824 | 1.072 |
| *Tetracladium marchalianum* | **11.774*** | **45.899***** | 0.594 |  | 4.667 | 13.437 | 4.043 |
| *Tetracladium sp.* | **13.629**** | **25.265**** | 0.015 |  | 4.940 | 11.523 | 3.298 |
| *Trechispora sp.* | **14.477**** | 5.080 | 0.049 |  | 2.823 | 0.870 | 3.001 |
| *Tulasnella sp.* | **9.838*** | 0.020 | 0.118 |  | 0.874 | 0.393 | 3.812 |
| *Umbelopsis sp.* | **12.847*** | 2.765 | 2.773 |  | 4.650 | 11.469 | 0.034 |
